# Prospective and comparative study of paroxysmal nocturnal hemoglobinuria patients treated or not by eculizumab: Focus on platelet extracellular vesicles

**DOI:** 10.1097/MD.0000000000016164

**Published:** 2019-07-05

**Authors:** Bérangère Devalet, Adeline Wannez, Nicolas Bailly, Lutfiye Alpan, Damien Gheldof, Jonathan Douxfils, Benoît Bihin, Bernard Chatelain, Jean-Michel Dogné, Christian Chatelain, François Mullier

**Affiliations:** a Department of Hematology, Namur Thrombosis and Hemostasis Center (NTHC), CHU UCL Namur, Université Catholique de Louvain, Yvoir; b Department of Pharmacy, Namur Thrombosis and Hemostasis Center (NTHC), University of Namur, Namur; c Hematology Laboratory, Namur Thrombosis and Hemostasis Center (NTHC), CHU UCL Namur, Université Catholique de Louvain, Yvoir; d Qualiblood s.a., Namur; e Scientific Support Unit, CHU UCL Namur, Université Catholique de Louvain, Yvoir, Belgium.

**Keywords:** eculizumab, extracellular vesicles, paroxysmal nocturnal hemoglobinuria, procoagulant activity, thrombosis

## Abstract

**Abstract:**

Thrombosis are severe complications of paroxysmal nocturnal hemoglobinuria (PNH), effectively reduced by eculizumab. Extracellular vesicles (EVs) may play a central role. The objective of this study was to assess the procoagulant activity of plasma isolated from PNH patients (treated or not by eculizumab) and to quantify their circulating EVs.

We iteratively collected the platelet-free-plasma of 17 PNH patients and 16 matched healthy volunteers, quantified their circulating EVs by flow cytometry and evaluated their procoagulant activity by thrombin generation and STA-Procoag-procoagulant phospholipid (PPL) assays.

A significant decrease of EVs from platelets (*P* = .024) and an increase of the STA-Procoag-PPL clotting time (*P* = .049) was observed after initiation of eculizumab and up to 11 weeks after. This reduction of prothrombotic biomarkers was not observed with the thrombin generation test due to a lack of sensitivity of this assay. Active hemolysis was observed in 90% of patients and elevated D-dimers in 41% of them. However, no significant difference was observed between patients and control subjects regarding the procoagulant activity, the EVs quantity, or the cellular origin. Lactate dehydrogenase (LDH) levels were lower in eculizumab-treated patients compared to nontreated patients (441 vs 2448 IU/L). D-dimers and LDH decreased after administration of eculizumab (mean decrease of 1307 ng/mL and 4159 IU/L, respectively).

These observations suggest a decrease of the phospholipid-dependent procoagulant potential of EVs after eculizumab therapy in PNH patients.

**Trial registration:**

NUB: B039201214365

## 1 Introduction

Paroxysmal nocturnal hemoglobinuria (PNH) is a rare, acquired disorder of the pluripotent hematopoietic stem cell due to an increased sensitivity toward attack of the complement, resulting of intravascular hemolysis. Venous and arterial thrombotic events are frequent and making the complications of the disease challenging to treat. Before the discovery and the generalization of eculizumab therapy, 40% to 67% of PNH patients died of thrombotic complications.^[1]^

The exact pathophysiology of thrombosis in PNH is still unknown but probably multifactorial and extracellular vesicles (EVs) may play a central role.^[2]^ In previous studies, increased levels of circulating EVs have been measured in PNH patients,^[3–7]^ a majority of them coming from platelets.^[3]^ Platelets EVs [extracellular vesicles of platelet origin (PEVs)] are known in vitro to have a 50- to 100-fold higher procoagulant activity than activated platelets.^[8]^ Eculizumab, a humanized monoclonal antibody directed against the complement protein C5, has demonstrated its efficacy in preventing the occurrence of thromboembolic events in PNH.^[9]^ The mechanisms involved remain misunderstood.

Our hypothesis is that eculizumab is able to prevent an excessive release of EVs in PNH patients, reducing their procoagulant potential.

The objective of this study is thus to assess the procoagulant activity of plasma isolated from PNH patients, treated or not by eculizumab, and to characterize their circulating EVs in terms of quantity and cellular origin. We aim at comparing the same parameters measured simultaneously in healthy subjects and in PNH patients to monitor the impact of eculizumab on these parameters in PNH patients.

## 2 Materials and methods

### 2.1 Study design and patients

This clinical trial was monocentric, prospective, and observational. It was approved by our central ethics committee (Commission d’Ethique Biomédicale Hospitalo-Facultaire de l’UCL) and was in accordance with the Helsinki Declaration. After informed consent, patients aged of 18 years or older and suffering from PNH with a PNH clone measured by flow cytometry of at least 5% were able to be included in the study. Patients could be treated or not by eculizumab at the time of inclusion. The inclusion period started in December 2012 and stopped in July 2014. Pregnant patients or patients who underwent hematopoietic stem cell transplantation were excluded.

Blood tests were performed at inclusion and repeated 6 months, 1 year, and 2 years later. The induction phase of eculizumab therapy consists of 4 infusions of 600 mg once a week, followed by a 900 mg infusion every 2 weeks in maintenance. For patients starting eculizumab during follow-up (decision left to the treating hematologist), the blood was collected just before the first infusion of 600 mg, at 5 weeks just before the first dose of 900 mg and at 11 ± 2 weeks during maintenance treatment.

At each inclusion of a PNH patient, a healthy volunteer of similar sex and age was simultaneously included in the study. Blood samples from PNH patient and healthy volunteer were analyzed together. These healthy volunteers were only seen at inclusion, should not suffer from any active disease and should not have a personal history of thrombosis. In addition, pregnancy, tobacco consumption, and treatment with antiplatelet agents, nonsteroidal anti-inflammatory drugs, or anticoagulants within 10 days before inclusion, were exclusion criteria.

PNH patients had to come to the Hematology Laboratory of CHU UCL Namur (Godinne site) in Belgium to undergo a blood test at each time point of the study. The same blood test was performed at inclusion for healthy volunteers. Clinical characteristics were collected for each PNH patient: age, sex, weight, height, and medical and surgical history including thrombosis, transfusion history, and drug treatment.

The blood was collected by venipuncture in the antecubital vein, in the morning, in a fasting state, after 30 minutes at rest, and at the hematology laboratory. The blood collection was performed in agreement with the recommendations of the International Society of Thrombosis and Hemostasis (ISTH) regarding the preanalytical conditions for EVs characterization.^[10]^

A portion of the collected blood served for routine hematology (Sysmex XN-9000, Kobe, Japan), chemistry analysis (VITROS 5600 Integrated System, Ortho Clinical Diagnostics, Raritan, NJ) and D-dimers quantification (Stago, Asnière-sur-Seine, France). PNH clone size was determined by analysis of neutrophils, monocytes, and red blood cells (RBC) in flow cytometry, in agreement with international guidelines.^[11,12]^ Glomerular filtration rate was estimated using the Modification of Diet in Renal Disease (MDRD) formula.

### 2.2 Platelet-free plasma and normal-pooled plasma

Three citrated tubes (Greiner bio-one, 3.5 mL, Sigma-Aldrich, Darmstadt, Germany) per patient or healthy volunteer were used for EVs analyses. According to the ISTH recommendations,^[10]^ the tubes were centrifuged at 2500 g for 15 minutes at room temperature. After this, the supernatant was pipetted and placed in a second tube, then centrifuged again under the same conditions. The second supernatant [platelet-free plasma (PFP)] was then pipetted, distributed in aliquots of 500 μL in low temperature freezer vials (VWR International bvba, Leuven, Belgium) and frozen without delay at −80°C. After thawing in a water bath at 37°C for 4 minutes, samples were used in the different experiments [for hemolysis measurement and characterization of EVs by flow cytometry, thrombin generation, and STA-Procoag-procoagulant phospholipid (PPL) assays]. It was previously demonstrated that deepfreeze storage conditions do not strongly influence EV analysis when performed adequately.^[13]^

It is important to note that a further concentration step of EVs by ultracentrifugation was later applied for thrombin generation assay (see below) but not for hemolysis measurement, flow cytometry, and STA-Procoag-PPL analysis. This was performed to find a solution to the lack of sensitivity of thrombin generation assay.^[14]^

The use of a normal pooled plasma (NPP) from other healthy volunteers was required to assess the impact of EVs on thrombin generation. For this purpose, the whole blood of 47 healthy subjects was collected on the same day by venipuncture. These samples were centrifuged twice at 2500 g during 15 minutes at 20°C. After pooling, the NPP was quickly frozen in liquid nitrogen and stored at −80°C.

### 2.3 Harboe's direct spectrophotometric method for hemolysis measurement

Two hundred microliters of each sample was placed in a cuvette (Sarstedt AG&Co., Nümbrecht, Germany), and 1800 μL of distilled water was added, giving a 10-fold dilution of the sample. A pipette was used for gentle mixing followed by resting for 10 minutes. The absorbance of each sample was read at 3 different wavelengths (380, 415, and 450 nm) against a distilled water blank in the spectrophotometer (Genesys 10S UV-Vis, Thermo Fisher Scientific Inc, Waltham, MA). The absorbance was converted into free hemoglobin concentration according to Han et al^[15]^ before calculating the degree of hemolysis. This last value was calculated by taking into account the hematocrit and total hemoglobin level.

### 2.4 Flow cytometry

Samples were analyzed on an FACS Aria flow cytometer with FACSDiva V6.1.3 software (BD Biosciences, San Jose, CA). The cytometer was optimized and programmed following the standardized procedures for measurement of EVs provided by the ISTH.^[16–18]^

The cytometer was calibrated with Megamix-Plus SSC beads (BioCytex, Marseille, France). These beads are optimized for use on cytometer FACS Aria.^[18]^ The separation index was used to evaluate the resolution on SSC parameter.^[19]^

The monoclonal antibodies used were CD235a-PE (IOTest, clone 11E4B-7-6, Beckman Coulter, Marseille, France), CD31-V450 (clone WM59, BD Biosciences, Erembodegem, Belgium), CD33-PE (clone P67.6, BD Biosciences), CD14-V450 (clone MϕP9, BD Biosciences), CD41a-PerCP-Cy5.5 (clone HIP8, BD Biosciences). Annexin V-FITC (BD Pharmingen, Erembodegem, Belgium) was used to tag phosphatidylserine present at EVs’ surface.

Before use, each monoclonal antibody was centrifuged at 15,000 g for 10 minutes at 4°C to remove antibodies aggregates. They were stored at 4°C to 6°C in the dark until use (within 4 hours). Samples were thawed in a water bath at 37°C for 4 minutes, just before incubation with antibodies, for 30 minutes at room temperature in the dark.

Then, the samples were introduced in the flow cytometer. The number of events during the acquisition period of 120 seconds was recorded. With an identical volume of sample and a constant flow during each analysis (5.4 μL/min), the absolute concentration of EVs could be calculated.

### 2.5 Calibrated automated thrombogram

The thrombin generation tests were performed on a Fluoroskan Ascent (Thermo Scientific, Waltham, MA) using the Thrombinoscope software (v 3.0, Thrombinoscope BV, Maastricht, The Netherlands). Each sample was run in triplicates.

#### 2.5.1 Concentration of EVs

To isolate EVs, samples were placed in a microcentrifuge polyallomer tube (Beckman Coulter, Marseille, France) and ultracentrifuged at 100,000 g for 90 minutes at 4°C. The pellet of EVs was suspended in a volume of supernatant to concentrate EVs 6.7 times more than that in the PFP. This further concentration step of EVs was needed to trigger thrombin generation assay without the addition of any reagents containing tissue factor (TF) or phospholipids. The tubes were spun for 1 minute and were stored on ice until being plated for calibrated automated thrombogram (CAT) analysis.

To evaluate the possible impact of TF bearing vesicles, anti-TF antibody (purified mouse anti-human CD142, BD Bioscience, Erembodegem, Belgium) was added to the concentrate of EVs at a final concentration of 10 μg/mL. This mixture was incubated during 10 minutes at 37°C.

#### 2.5.2 Thrombin generation assay

Twenty microliter of EVs concentrate, 20 μL of EVs concentrate with anti-TF, and 20 μL of the supernatant of EVs concentrate were tested. Twenty microliter of thrombin calibrator was added to 3 other wells. For interplate quality control, 3 wells contained 20 μL of PPP-Reagent Low (Thrombinoscope BV), constituted of TF and phospholipids.

The plate was then incubated for 10 minutes in the CAT system at 37°C and during this time, the NPP was thawed in a water bath at 37°C. Afterwards, 80 μL of NPP was added to each well and 20 μL of FluCa (mix of fluo-buffer and fluo substrate) (Thrombinoscope BV) were added by the dispenser.

### 2.6 STA-Procoag-PPL

The STA-Procoag-PPL kit (Diagnostica Stago SAS, Asnières-sur-Seine, France) is designed for the detection of PPLs in plasma samples by a chronometric method. This assay measures clotting time, in the presence of factor Xa and CaCl_2_, of a system in which all the factors are present at physiological levels (supplied by PPL depleted plasma) except the PPL supplied by the plasma sample being tested.^[20]^

Five hundred microliters of each PFP sample was placed in a cuvette and introduced in a STA-R Evolution coagulometer (Diagnostica Stago SAS).

### 2.7 Statistical analysis

Quantitative data are described with median and range. *P* values are computed with the Wilcoxon signed rank test or Wilcoxon rank sum test for independent and paired data, respectively. Statistical analyses are performed with R 3.3.2 software (R Foundation for Statistical Computing, Vienna, 2016).

## 3 Results

### 3.1 Patients’ characteristics

After informed written consent, 17 PNH patients and 16 healthy volunteers were included in the study. It was not possible to find a sex/age-matched healthy volunteer to include simultaneously with patient 14. At inclusion, 7 of the 17 PNH patients (P02, P04, P06, P08, P10, P13, and P15) were chronically treated by eculizumab. Four more patients (P01, P05, P12, and P14) started eculizumab therapy during follow-up. The sex ratio M/F was 5/12. The median age of PNH patients was 44 (range: 22–79) years. A personal history of thrombosis was reported in 6 patients: 2 portal vein thrombosis (P05 and P12), including 1 with pulmonary embolism (P05), 2 deep vein thrombosis (P13 and P17), and 2 superficial vein thrombosis (P08 and P11) (Table 1). Three patients were treated by low-dose corticosteroids (P01, P10, P14), 2 patients by low-dose acetylsalicylic acid (P03, P11), and 3 patients by vitamin K antagonists (P09, P12, and P13). The median PNH clone was 93.0% on neutrophils (min 22.5%, max 99.7%), 92.5% on monocytes (min 0.1%, max 99.8%), and 41.4% on RBC (min 4.1%, max 68.1%).

**Table 1:**
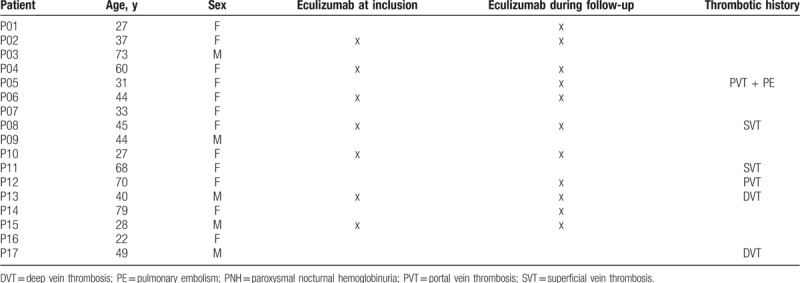
Demographic and clinical characteristics of study paroxysmal nocturnal hemoglobinuria patients.

### 3.2 Impact of eculizumab therapy on PNH patients

**Figure 1:**
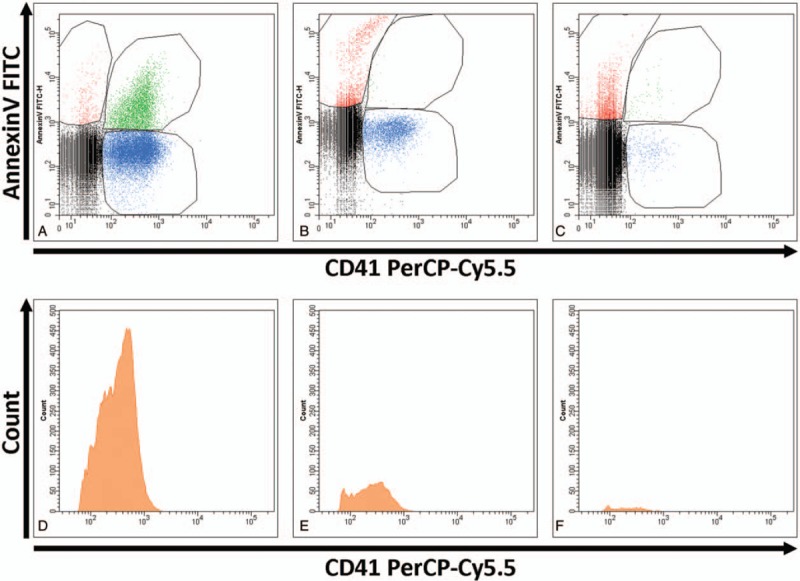
Decrease of the number of platelet-derived extracellular vesicles detected by flow cytometry in patient 1 before and after eculizumab treatment initiation. A, B, and C, The extracellular vesicles of platelet origin (PEVs) detected just before eculizumab treatment initiation (A), at 5 weeks just before the first dose of 900 mg (B), and at 11 weeks during maintenance treatment (C) are shown according to their labeling by annexin V and anti-CD41. Annexin V negative PEVs are shown in blue and annexin V positive PEVs in green. A first than a second decrease of all PEVs is observed after initiation of eculizumab. D, E, and F, The total of PEVs detected before eculizumab (D), at 5 weeks (E), and 11 weeks after treatment initiation (F) are shown according to their count and their labeling of CD41.

**Figure 2:**
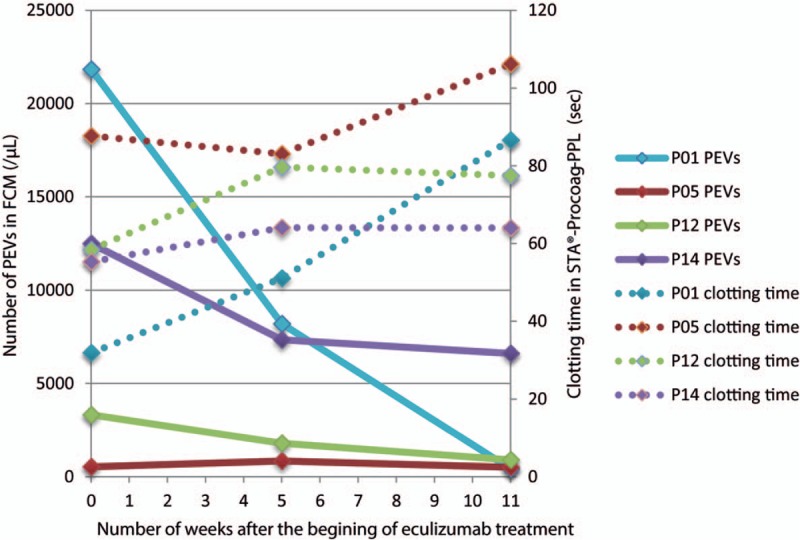
Evolution of PEVs (flow cytometry) and clotting time (STA-Procoag-PPL) after eculizumab treatment in PNH patients. This figure illustrates the evolution of the number of PEVs detected by flow cytometry (full lines) and the clotting time measured in STA-Procoag-PPL assay (dotted lines) after the start of eculizumab. Eculizumab was administered just after the first blood sample. An evolution in mirror of these 2 parameters can be observed. PEV = extracellular vesicle of platelet origin; PNH = paroxysmal nocturnal hemoglobinuria.

**Figure 3:**
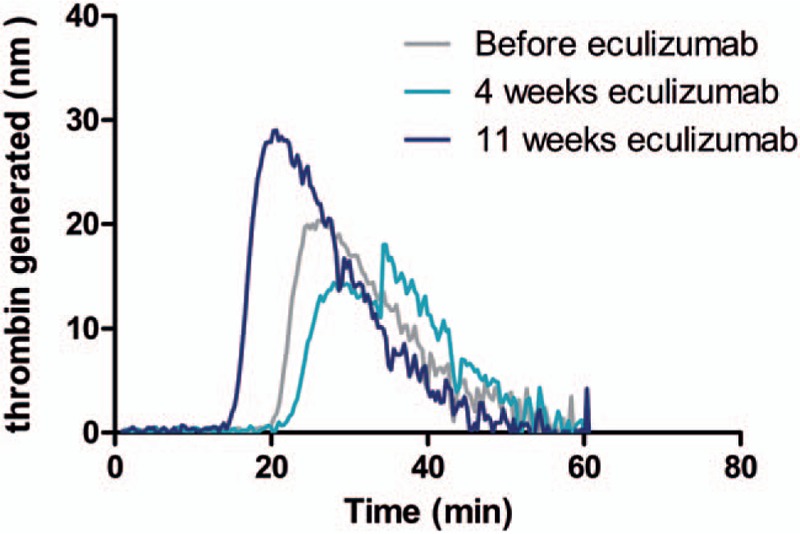
Evolution of the amount of thrombin generated (CAT curves) before and after eculizumab treatment in paroxysmal nocturnal hemoglobinuria (PNH) patient 14. This figure illustrates, in patient 14, the evolution of the amount of thrombin generated during the reaction time with CAT before eculizumab (in grey), 4 weeks (in light blue), and 11 weeks (in dark blue) after starting treatment. Even if a slight decrease of the lagtime and an increase of the area under the curve (endogeneous thrombin potential) were observed after 11 weeks of eculizumab, this was not observed consistently in the other 3 patients. CAT = calibrated automated thrombogram.

We observed a significant decrease of EVs of platelet origin (PEVs, defined as CD41+ EVs in flow cytometry) after initiation of eculizumab therapy (*P* = .024). A first decrease was observed after 4 weeks of eculizumab therapy (mean −5003 PEVs/μL), which corresponds to the end of the induction treatment phase, and a second decrease after 11 ± 2 weeks of treatment (mean change from baseline −7352 PEVs/μL). A parallel evolution of annexin V positive (+) PEVs and annexin V negative (−) PEVs was observed. This is illustrated in Figure 1. There was no clear tendency in the evolution of the other subgroups of EVs. Along with this, we observed during the study a significant increase of the STA-Procoag-PPL clotting time in the group that has started eculizumab therapy compared to the group of nontreated patients (*P* = .049). The mean increase in the STA-Procoag-PPL clotting time was 11.2 seconds at 4 weeks and 27.8 seconds at 11 weeks (Fig. 2). However, this tendency was not observed in any of the 4 parameters measured with the thrombin generation assay (data not shown), as illustrated in Figure 3. A reduction of D-dimers levels was observed after the induction phase of treatment (mean decrease of 1307 ng/mL).

In addition, neutrophils and platelets counts were found to decrease after initiation of eculizumab therapy. As expected, hemoglobin and hematocrit levels increased while free hemoglobin and reticulocytes levels decreased. Simultaneously, lower levels of lactate dehydrogenase (LDH) were measured (mean decrease of 4159 IU/L), indicating a reduction in hemolysis.

PNH clone size was not significantly impacted by initiation of eculizumab therapy when measured on neutrophils and monocytes but there was a tendency to an increase of PNH clone measured on RBC.

### 3.3 Comparison of PNH patients not treated by eculizumab and their matched controls

Routine hematology and chemistry analysis revealed some significant differences in PNH patients not treated by eculizumab compared with healthy volunteers (Table 2). Lower levels of platelets and lymphocytes were observed in untreated PNH patients than in control subjects (about 2-fold decrease). Regarding to hemolysis parameters, lower hemoglobin and hematocrit levels and higher LDH and reticulocytes levels were observed in PNH patients compared to controls. Moreover, the haptoglobin level was not measurable (<3.5 mg/dL) in 89% of untreated PNH patients, whereas it was never lowered in control subjects. The free hemoglobin concentration was higher in PNH patients than in control subjects. Renal function was not different in PNH patients compared to controls.

**Table 2:**
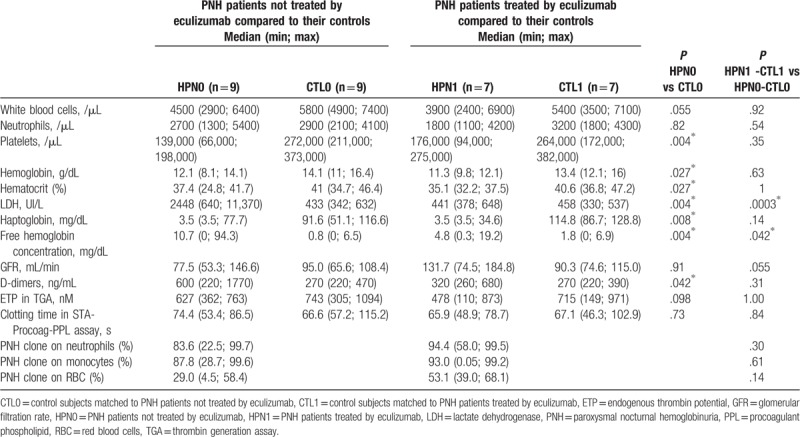
Description of measured parameters for paroxysmal nocturnal hemoglobinuria patients treated or not by eculizumab and their matched controls.

In terms of procoagulant activity, D-dimers were elevated (>500 ng/mL) in 66.7% of the untreated PNH patients but in none of the controls. However, the procoagulant activity measured by either thrombin generation or STA-Procoag-PPL assays was not significantly different between untreated PNH patients and control subjects.

It is important to note that variability was observed in results obtained within the triplicates in thrombin generation assay (mean interwells coefficient of variation: 15.7%). Moreover, the addition of anti-TF antibody to the wells was associated with a lengthening of the lagtime in only 37% of the control subjects and in 29% of PNH patients.

The EVs measured in flow cytometry did not differ significantly in term of quantity or surface antigens in untreated PNH patients and control subjects.

### 3.4 Comparison of PNH patients treated by eculizumab and their matched controls

Globally, the differences observed between treated PNH patients and their controls appeared similar to those observed between untreated PNH patients and their relative control, except for LDH, free hemoglobin, and D-dimer levels (Table 2).

The haptoglobin level was not measurable in 91.7% of treated PNH patients, while always normal in control subjects. However, the LDH levels measured in treated patients were comparable to controls. Free hemoglobin concentration increased less in treated patients than in untreated patients. D-dimers were elevated in only 16.7% of the eculizumab treated PNH patients compared to 66.7% in untreated patients.

Of note, changes in thrombin generation parameters, clotting time in STA-Procoag-PPL and characteristics of EVs between treated PNH patients were similar to the ones observed between untreated PNH patients and matched controls.

The PNH clone measured on neutrophils or monocytes was similar in eculizumab treated and not treated PNH patients. More relevant differences were observed on RBC, with a mean of 53.1% in treated patients versus 29.0% in nontreated patients.

## 4 Discussion

In this prospective and comparative study of PNH patients, we observed a significant decrease of the number of PEVs measured by flow cytometry after initiation of eculizumab therapy, along with an increase of the STA-Procoag-PPL clotting time. This may reflect a decrease of the phospholipid-dependent procoagulant potential of the EVs isolated from plasma of PNH patients after eculizumab therapy.

Van Bijnen et al^[21]^ suspected a higher level of platelet activation induced by complement in PNH, by the fact that PNH platelets, after membrane attack complex stimulation, expose more FVa-binding sites and increase thrombin generation. Platelet activation is a well-known process leading to the release of procoagulant PEVs.^[22–24]^ In our study, PNH patients chronically treated by eculizumab or not treated at all do not show a significant difference in term of PEVs quantity or phospholipid-dependent procoagulant activity compared to normal subjects. These observations may be attributed to an interindividual variability of the number of circulating EVs in general population and in pathological states^[23]^ and the small number of patients included in our study. However, if we focus on each PNH patient who starts eculizumab therapy, the decrease of their number of PEVs and of their phospholipid-dependent procoagulant profile appears in 3 of the 4 patients (P1, P12, P14). The remaining patient (P5) had already a low level of PEVs before eculizumab therapy. The decrease of the number of PEVs occurs very quickly after the induction phase of treatment, due to the dose intensity of eculizumab, and seems to continue to decrease steadily during the maintenance phase of treatment. Weitz et al^[7]^ observed, in their study on 11 PNH patients, a decrease of sP-selectin level after eculizumab therapy. This provides additional argument for a decrease of platelet (and endothelial) activation induced by complement inhibition. Platelet activation remains difficult to study, as long as a reliable plasma marker of platelet activation has not been identified.^[25]^ More studies are needed to evaluate the potential of PEVs as marker of platelet activation in PNH.

Moreover, we observed an increased level of D-dimers in PNH patients compared to control subjects and a decrease of this marker after initiation of eculizumab therapy. This is in agreement with previous studies,^[7,26–28]^ confirming that D-dimers may be a marker of hemostatic activation in PNH, even if its lack of specificity remains a problem.

Biological parameters confirm active hemolysis in most of PNH patients included in our study. A higher LDH level was observed in patients compared to control and it decreased during eculizumab therapy. A retrospective analysis of 301 patients from the South Korean National PNH Registry showed that PNH patients with elevated LDH levels (≥1.5 the upper limit of normal) had a significantly higher risk of thromboembolic event (odds ratio: 7.0; *P* = .013).^[29]^ The link between hemolysis and thrombosis in PNH is still controversial^[2]^ but eculizumab clearly demonstrated its efficacy to reduce hemolysis.^[30,31]^

In this study, contrary to what we expected, we did not observe an increase of thrombin generation in PNH patients compared to control subjects, neither a decrease of this profile after eculizumab therapy. The lack of sensitivity of thrombin generation assay and the variability of results obtained may limit the possibility to detect tight variations between these groups, which are small and heterogeneous.^[14,32]^ The addition of small amounts of phospholipids and TF may facilitate the reaction and increase its sensitivity.^[33]^ Moreover, the use of anti-TF pathway inhibitor may help to reduce the intrinsic variability of this assay.^[14]^ Finally, we could not totally exclude an impact of free hemoglobin, released by hemolysis, on the amount of thrombin generated in this assay. In this fluorogenic assay, free hemoglobin may increase the absorbance of the sample. Previous studies demonstrated that free plasma hemoglobin was able to falsely reduce thrombin peak height in thrombin generation assays, in direct proportion to the concentration of hemoglobin in the sample.^[27,34]^ However, the use of thrombin calibrator, as we did in this study, minimizes this impact.^[34]^

An increased level of TF-bearing microparticles in PNH patients, decreasing after eculizumab therapy, was previously described but this was not correlated to changes in TF microparticle activity or total microparticle factor Xa generation.^[7]^ In our study, the addition of anti-TF antibodies did not affect thrombin generation results more significantly in PNH patients than in control subjects, reinforcing the assumption that most of the TF on the circulating EVs in PNH patients is encrypted and nonfunctional.^[7]^

In conclusion, the simultaneous decrease of the number of PEVs and of the phospholipid-dependent procoagulant activity that we observed in the plasma of PNH patients newly treated by eculizumab may partially explain the dramatic reduction of thrombotic risk obtained by this treatment. However, this needs to be further studied. We did not observe any significant difference in term of EVs or procoagulant activity (excepted D-dimers) between PNH patients and healthy subjects or between chronically treated and not treated PNH patients by eculizumab. Similarly, no significant difference was observed between patients who previously experienced thrombosis and who did not. Unfortunately, our study, like most prospective studies carried on PNH patients, suffers from a lack of statistical power because of the small number and the heterogeneity of patients studied (including treatment or not by antithrombotic agents) and the rarity of thrombotic events during a short follow-up period. The observation of the impact of eculizumab on clinical and biological parameters of each patient individually, before and after initiation of treatment, deserves to be further extended. Today, it is very hazardous to make connections between biological observations and clinical risk. Nevertheless, development of biomarkers able to predict thrombotic risk is needed and the idea of PEVs in this role is attractive.

## Author contributions

BD and FM designed the research study. BD, AW, NB, and LA performed the research. BD analyzed the data and wrote the article. DG, JD, BC, FM, and JMD contributed essential reagents and tools. BB performed the statistical analysis. BD, AW, BC, CC, and FM interpreted the data. All authors revised the article.

**Conceptualization:** Bérangère Devalet, François Mullier.

**Data curation:** Bérangère Devalet, Adeline Wannez.

**Formal analysis:** Bérangère Devalet, Adeline Wannez, Benoît Bihin, François Mullier.

**Funding acquisition:** Bérangère Devalet, Christian Chatelain.

**Investigation:** Bérangère Devalet, Adeline Wannez, Nicolas Bailly, Lutfiye Alpan, Bernard Chatelain, Jean-Michel Dogné, Christian Chatelain.

**Methodology:** Bérangère Devalet, Damien Gheldof, Jonathan Douxfils, Jean-Michel Dogné, François Mullier.

**Project administration:** Bérangère Devalet.

**Resources:** Damien Gheldof, Jonathan Douxfils, Jean-Michel Dogné.

**Supervision:** François Mullier.

**Validation:** Bérangère Devalet, Adeline Wannez, Bernard Chatelain, Jean-Michel Dogné, François Mullier.

**Writing – original draft:** Bérangère Devalet.

**Writing – review and editing:** Bérangère Devalet, Adeline Wannez, Nicolas Bailly, Lutfiye Alpan, Damien Gheldof, Jonathan Douxfils, Benoît Bihin, Bernard Chatelain, Jean-Michel Dogné, Christian Chatelain, François Mullier.
